# Population Access to US Trauma Centers and Teletrauma-Using Emergency Departments

**DOI:** 10.1001/jamanetworkopen.2025.56958

**Published:** 2026-02-16

**Authors:** Zain G. Hashmi, Russell Griffin, Janice A. Espinola, Ashley F. Sullivan, Krislyn M. Boggs, Maeve Swanton, Molly P. Jarman, Jan O. Jansen, Jeffrey D. Kerby, Carlos A. Camargo

**Affiliations:** 1 Division of Trauma and Acute Care Surgery, Department of Surgery, University of Alabama at Birmingham, Birmingham; 2 Center for Injury Science, University of Alabama at Birmingham, Birmingham; 3 Department of Emergency Medicine, Massachusetts General Hospital, Boston; 4 Center for Surgery and Public Health and Department of Surgery Brigham and Women’s Hospital, Harvard Medical School, Harvard T.H. Chan School of Public Health, Boston, Massachusetts; 5 Harvard Medical School, Boston, Massachusetts

## Abstract

This cross-sectional study determines the additional population access to trauma care expertise provided by telehealth for trauma in emergency departments and provides updated national estimates of trauma center access.

## Introduction

Trauma centers (TCs) are specialized hospital units with resources and expertise to treat injured patients. In the US, a hierarchical network of TCs exists.^1^ Levels 1 and 2 (ie, advanced) TCs provide definitive injury management, while level 3 and lower (ie, basic) TCs offer initial care and often transfer patients to advanced TCs.

Prior research suggests that 23% of the US population and 42% of the rural population lack access to TC care within 60 minutes by ground ambulance, a disparity that contributes to worse outcomes.^2^ Although building new TCs can improve access, this strategy is cost- and resource-prohibitive.^3,4^ Teletrauma (clinician-to-clinician telehealth for injury care) has emerged as a promising alternative to expand access to trauma care expertise, but its geographic reach beyond existing TCs remains unclear.^5,6^ We aimed to determine the additional population access to trauma care expertise provided by teletrauma-using emergency departments (TTEDs) and provide updated national estimates of TC access.

## Methods

This cross-sectional study was approved by the Mass General Brigham Institutional Review Board with a waiver of informed consent owing to non–human participant research. We followed the STROBE reporting guideline.

Using the 2022 National Emergency Department Inventory (NEDI)-USA, we identified all adult TCs and self-reported TTEDs (>80% response). The unit of analysis was the 2020 US Census block group (CBG), with location of injury defined as the population-weighted centroid. We defined access as the highest-level TC (advanced TCs highest and TTEDs lowest) available to each CBG within 60 minutes by ground ambulance. Network cost analysis (ArcGIS Pro, version 3.4.3; ESRI) estimated drive times as the sum of emergency medical services (EMS) dispatch, departure, and scene times (per 2023-2024 data from the National EMS Information System) and travel time from EMS base to centroid to facility. Sensitivity analyses including air EMS transport were performed (eMethods in Supplement 1). Urbanicity was defined using 2013 Urban Influence Codes. Additional details, including methodological limitations, are provided in eMethods in Supplement 1.

## Results

In 2022, 4607 EDs responded to NEDI-USA; 379 (8%) were TTEDs. Among TTEDs, 347 (92%) were located at facilities without a TC. Among 1505 EDs in rural areas without a TC, 286 (19%) reported using teletrauma.

Of 331 351 858 people in the US population, an estimated 264 million (79.7%) had access to a TC within 60 minutes by ground ambulance. Although 40 748 183 people (13%) had access to a TTED, these facilities expanded access to trauma care by only 2% (7.3 million) (Table and Figure). TTEDs provided access to 10% of 67 214 203 people without prior access to any TC. This proportion varied regionally: 2 506 209 of 13 760 768 (18%) in the Midwest, 1 081 095 of 8 835 829 (12%) in the Northeast, 2 938 959 of 33 227 024 (9%) in the South, 771 650 of 11 390 582 (7%) in the West, and 4 748 473 of 33 783 378 (14%) in rural areas.

**Table.** Estimated Population Access to Trauma Care Expertise in the US Within 60 Minutes by Ground Emergency Medical Services Transport

Population	Population in millions, No. (%)^a^			
	Advanced trauma center^b^	Basic trauma center^c^	TTEDs	No access to trauma care expertise
Total	235.0 (71)	29.1 (9)	7.3 (2)	60.0 (18)
Urbanicity^d^				
Rural	4.0 (9)	8.2 (18)	4.7 (10)	29.0 (63)
Urban	231.0 (81)	21.0 (7)	2.5 (1)	30.9 (11)
Census regions				
Midwest	48.1 (70)	7.1 (10)	2.5 (4)	11.3 (16)
Northeast	46.8 (81)	2.0 (3)	1.1 (2)	7.8 (14)
South	78.7 (62)	14.3 (11)	2.9 (2)	30.3 (24)
West	61.4 (78)	5.7 (7)	0.8 (1)	10.6 (14)

Abbreviations: TTED, teletrauma-using emergency department.

^a^ Describes mutually exclusive, tiered access to highest level of trauma care expertise available within 60 minutes by ground emergency medical services transport.

^b^ American College of Surgeons–similar level 1 or 2 adult trauma center.

^c^ American College of Surgeons–similar level 3 adult trauma center, defined using the US Department of Agriculture 2013 Urban Influence Codes.

^d^ Micropolitan and noncore regions were classified as rural and the remainder were classified as urban.

**Figure.**
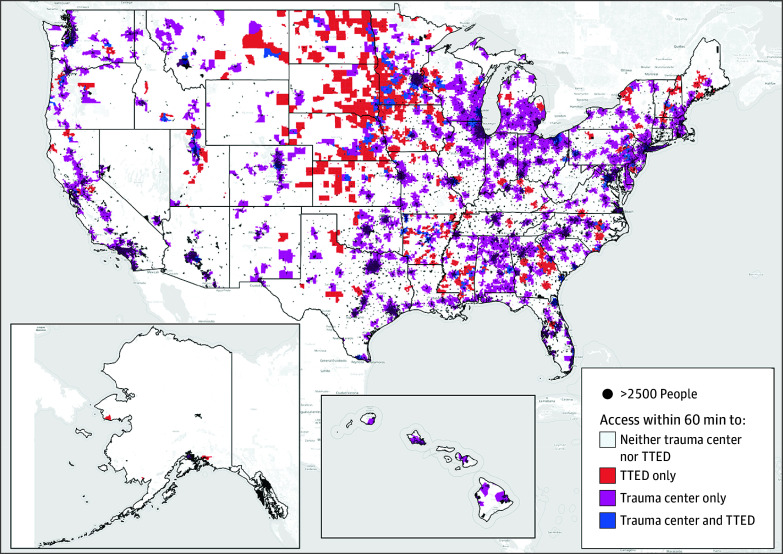
Map Depicting Estimated Population Access to Trauma Care Expertise in the US Within 60 Minutes by Ground Emergency Medical Service Transport TTED indicates teletrauma-using emergency department.

Approximately 60 million people (18% of the US population) lacked access to any level of trauma care, more than half (30 288 365 [51%]) of whom resided in the South. Air EMS sensitivity analysis accounted for additional trauma care access to 317 045 people (0.1%).

## Discussion

TTEDs improve access to trauma care expertise for 1 in 10 people who previously lacked access to a TC. Most TTEDs are located near existing TCs, limiting their impact on underserved areas. Our updated national estimates align with 2019 data and confirm that millions of US residents still lack timely trauma care access.

A key limitation of this study is the self-reported use of teletrauma on the survey; however, the survey was completed by ED directors who likely had correct information. Our findings suggest that TTEDs are not being strategically deployed to expand access to trauma care, especially in rural areas. Targeted efforts are needed to expand access to teletrauma care to realize its true potential in alignment with goals established by the American College of Surgeons Committee on Trauma.^5^

## Notes

Supplement 1. **eMethods.** Identification of Trauma Centers and Teletrauma-Using Emergency Departments, Travel Time and Population Access, and Sensitivity Analysis
eReferences

Supplement 2.
Data Sharing Statement
