# Supplementary material for: Population Access to US Trauma Centers and Teletrauma-Using Emergency Departments
Source: JAMA Netw Open. 2026 Feb 16;9(2):e2556958. doi: 10.1001/jamanetworkopen.2025.56958 (PMC12910394; doi:10.1001/jamanetworkopen.2025.56958)
Supplement: Supplement 1. — eMethods. Identification of Trauma Centers and Teletrauma-Using Emergency Departments, Travel Time and Population Access, and Sensitivity Analysis eReferences [file jamanetwopen-e2556958-s001.pdf]

Supplementary Online Content

Hashmi ZG, Griffin R, Espinola JA, et al. Population access to trauma centers and teletrauma-using emergency departments. *JAMA Netw Open.* 2026;9(2):e2556958. doi:10.1001/jamanetworkopen.2025.56958

eMethods. Identification of Trauma Centers and Teletrauma-Using Emergency Departments, Travel Time and Population Access, and Sensitivity Analysis
eReferences

This supplementary material has been provided by the authors to give readers additional information about their work.

© 2026 Hashmi ZG et al. *JAMA Network Open*.

eMethods. Identification of Trauma Centers and Teletrauma-Using Emergency Departments, Travel Time and Population Access, and Sensitivity Analysis

This study follows the Strengthening the Reporting of Observation Studies in Epidemiology (STROBE) reporting guidelines for cross-sectional studies.

Additional details of Emergency Department (ED) sample, identification of teletrauma-using EDs (TT-EDs) and Trauma Center (TC) status

We used the 2022 National ED Inventory (NEDI)-USA to identify all US EDs that were open in 2022. NEDI-USA is a long-standing, annual nationwide survey of all non-federal, non-specialty US EDs including hospital-based, satellite freestanding and autonomous freestanding EDs coordinated by the Emergency Medicine Network[1,2]. EDs were included if they were operational 24 hours/day, 7 days/week during 2022. The overall response rate for the 2022 survey was 82%. EDs with missing survey responses or responses indicating no telehealth service use were classified as not using teletrauma service.

TT-EDs were identified based on a two-part self-reported response to the NEDI-USA question: *"Does your ED utilize telehealth services to evaluate patients in your ED?"* and if *"YES, (check all that apply)"* with *"trauma"* as a listed option.

TCs are hospital-based units with varying resources and expertise to treat injured patients. Depending on the capabilities, TC status can range from Level 1 or 2 (more advanced capabilities) to Level 3 or lower (less advanced or basic capabilities). TC status is *designated* by the state government and/or *verified* by the American College of Surgeons (ACS). For this analysis, we classified TCs as basic or advanced based on our prior work that unified ACS verified and state designated trauma center levels[3]. ACS-similar levels 1 and 2 TCs were classified as advanced and ACS-similar level 3 TCs were classified as basic TCs. Using this approach, we determined the trauma care capability of each ED – whether the ED was located in an advanced or basic TC, reported using teletrauma service or had no specific trauma care

© 2026 Hashmi ZG et al. *JAMA Network Open*.

capability (i.e., no access to trauma care expertise either via a TC or the use of teletrauma). No EDs had missing TC status. Geocoordinates for each facility were available through NEDI-USA.

Additional Details of Travel Time Calculations and Population Access Analysis

We estimated the highest-level of trauma care facility available to each 2020 US census block group (CBG) within 60 minutes by ground ambulance. The US Census Bureau defines a CBG as "statistical divisions of census tracts…generally defined to contain between 600 and 3,000 people, and are used to present data and control block numbering[4]." Location of injury was defined as population-weighted centroid for each CBG. For this analysis, advanced TCs were considered as the highest-level trauma care facility, followed by basic TCs and TT-EDs, respectively. Time to a trauma care facility was defined as the sum of emergency medical services (EMS) dispatch, departure and scene times and travel time from EMS base to centroid and from centroid to facility. Median EMS dispatch (2.6 minutes), departure (1.4 minutes) and scene time (18 minutes) were obtained from actual ground EMS transport data using the National EMS Information System (NEMSIS) online "Data Cube" for year 2023-2024[5]. Network cost analysis (ArcGIS Pro, ESRI) considered all ground EMS bases and trauma care facilities within 500 km of each CBG centroid and estimated fastest possible driving routes accounting for existing traffic control measures (signals, stop signs, and directional restrictions). These data were then used to determine the highest-level of trauma care facility available to each CBG within 60 minutes and the total population associated with each level of access. For example, if the drive time to a TT-ED from a given centroid was 46 minutes and to a trauma center from the same centroid was 75 minutes, the highest level of trauma care facility available to the CBG represented by this centroid was the TT-ED.

We also evaluated the results stratified by urbanicity (urban/rural) and US Census divisions. Urbanicity was defined using the US Department of Agriculture 2013 Urban Influence

© 2026 Hashmi ZG et al. *JAMA Network Open*.

Codes where micropolitan and noncore regions were classified as rural and the remained of the regions were classified as urban[6].

The black dot for >2500 persons (Figure) were obtained from US Census Populated Place Areas which represent geographic polygons containing population totals from census data[7].

One potential limitation of the current analysis is the use of population-weighted centroids in place of incident locations. It is possible that the centroid is not representative of the true geographic location of injury-associated incidents requiring emergency care. However, nearly 90% of injuries occur within 10 miles of a person's residence (median 0.2 miles)[8]. Therefore we believe it is reasonable to consider the population-weighted centroid as the presumed injury incident location such that drive times estimated using actual injury incident location would not be significantly different. Additionally, this approach is also widely accepted within the trauma systems research community to map geographic access to trauma centers[9,10]. Another limitation is the use of a static rather than a dynamic model with consideration of time-delay variables, such as traffic congestion and weather. For example, it is possible that the currently estimated access areas are larger in size than if the traffic density were accounted for in the network cost analysis (i.e. an overestimation of population access). Dynamic models are important to consider when estimating access in local/smaller geographic areas. However, the real-world impact of traffic congestion at the national scale is likely small given that it is mostly limited to peak hours and urban areas. Therefore, most studies assessing nationwide access to trauma center care consider static models, while reserving dynamic models for local/smaller geographic areas[9–11].

© 2026 Hashmi ZG et al. *JAMA Network Open*.

Details of Sensitivity Analysis using Air EMS Transport

Geospatial data of air AMS base locations were obtained from the Emergency Transport Healthcare Operations and Safety (ETHOS) database[12]. We used the methodology previously described by Jarman et al to determine access to the highest-level of trauma care facility available to each CBG within 60 minutes by air ambulance[10]. Time to a trauma care facility was defined as the sum of emergency medical services (EMS) dispatch, departure and scene times and travel time associated with straight-line distance from air EMS base to centroid and from centroid to facility. Travel speed was set at 120 miles per hour which has previously been described as the average velocity of rotary-wing air ambulances in the US[13]. Median EMS dispatch (12.9 minutes), departure (5.3 minutes) and scene time (33.6 minutes) were obtained from actual air EMS transport data using the National EMS Information System (NEMSIS) online “Data Cube” for year 2023-2024[5]. These data were then combined with the ground transport data to determine highest-level TC facility within 60-minutes by either ground or air EMS.

© 2026 Hashmi ZG et al. *JAMA Network Open*.

eReferences

1. National Emergency Department Inventories (NEDI) – Emergency Medicine Network. Accessed September 23, 2025. https://www.emnet-usa.org/research/studies/nedi/

2. Sullivan AF, Richman IB, Ahn CJ, et al. A Profile of US Emergency Departments in 2001. *Annals of Emergency Medicine*. 2006;48(6):694-701. doi:10.1016/j.annemergmed.2006.08.020

3. Bedell BR, Boggs KM, Espinola JA, et al. Development of a unified national trauma center database, 2018. *Injury*. 2023;54(2):461-468. doi:10.1016/j.injury.2022.11.054

4. US Census Bureau. US Census Bureau Glossary. November 28, 2025. Accessed November 28, 2025. https://www.census.gov/programs-surveys/geography/about/glossary.html

5. National Highway Traffic Safety Administration. EMS Data Cube. NEMSIS. Accessed August 21, 2024. https://nemsis.org/view-reports/public-reports/ems-data-cube/

6. US Department of Agriculture. Urban Influence Codes | Economic Research Service. Accessed August 21, 2024. https://www.ers.usda.gov/data-products/urban-influence-codes

7. USA Census Populated Place Areas - Overview. Accessed November 29, 2025. https://www.arcgis.com/home/item.html?id=d8e6e822e6b44d80b4d3b5fe7538576d

8. Haas B, Doumouras AG, Gomez D, et al. Close to home: An analysis of the relationship between location of residence and location of injury. *J Trauma Acute Care Surg*. 2015;78(4):860-865. doi:10.1097/TA.0000000000000595

9. Carr BG, Bowman AJ, Wolff CS, et al. Disparities in access to trauma care in the United States: A population-based analysis. *Injury*. 2017;48(2):332-338. doi:10.1016/j.injury.2017.01.008

10. Jarman MP, Dalton MK, Askari R, Sonderman K, Salim A, Inaba K. Accessibility of Level III trauma centers for underserved populations: A cross-sectional study. *J Trauma Acute Care Surg*. 2022;93(5):664-671. doi:10.1097/TA.0000000000003725

11. Stolarski AE, Smith SM, Poulson M, et al. Equity of Access to Care in an Urban Trauma System. *Journal of Surgical Research*. 2025;314:298-304. doi:10.1016/j.jss.2025.07.010

12. Prehospital Resource Optimization and Delivery of Care in Trauma Systems (PRODCTS) lab, University of Pittsburgh. Emergency Transport Healthcare Operations and Safety (ETHOS) database. Accessed April 1, 2025. https://www.ethosdatabase.org/

13. Branas CC, MacKenzie EJ, ReVelle CS. A trauma resource allocation model for ambulances and hospitals. *Health services research*. 2000;35(2):489-507.

© 2026 Hashmi ZG et al. *JAMA Network Open*.
