# Supplementary material for: Population Access to US Trauma Centers and Teletrauma-Using Emergency Departments
Source: JAMA Netw Open. 2026 Feb 16;9(2):e2556958. doi: 10.1001/jamanetworkopen.2025.56958 (PMC12910394; doi:10.1001/jamanetworkopen.2025.56958)
Supplement: Supplement 2. — Data Sharing Statement [file jamanetwopen-e2556958-s002.pdf]

Data Sharing Statement

Hashmi. Population Access to US Trauma Centers and Teletrauma-Using Emergency Departments. *JAMA Netw Open*. Published February 16, 2026.
doi:10.1001/jamanetworkopen.2025.56958

Data
Data available: Yes
Data types: Other (please specify)
Additional Information: Partial National Emergency Department Inventory-USA data, the data dictionary, and analytic code are immediately available on reasonable request to CAC (ccamargo@partners.org).
How to access data: Partial National Emergency Department Inventory-USA data, the data dictionary, and analytic code are immediately available on reasonable request to CAC (ccamargo@partners.org).
When available: With publication

Supporting Documents
Document types: None

Additional Information
Who can access the data: researchers whose proposed use of the data has been approved
Types of analyses: for a specified purpose
Mechanisms of data availability: with investigator support, after approval of a proposal and with a signed data access agreement
